# MRI evaluation of foraminal changes in the cervical spine with assistance of a novel compression device

**DOI:** 10.1038/s41598-023-38401-5

**Published:** 2023-07-17

**Authors:** J. Hutchins, K. Lagerstrand, E. Stävlid, P-A. Svensson, K. Rennerfelt, H. Hebelka, H. Brisby

**Affiliations:** 1 Institute of Clinical Sciences, Sahlgrenska Academy, University of Gothenburg, Gothenburg, Sweden; 2 Department of Orthopaedics, Sahlgrenska University Hospital, Gothenburg, Sweden; 3 Department of Radiology, Sahlgrenska University Hospital, Gothenburg, Sweden; 4 Department of Medical Physics and Biomedical Engineering Physics, Sahlgrenska University Hospital, Gothenburg, Sweden

**Keywords:** Medical research, Preclinical research

## Abstract

Standard supine Magnetic Resonance Imaging (MRI) does not acquire images in a position where most patients with intermittent arm radiculopathy have symptoms. The aim of this study was to test the feasibility of a new compression device and to evaluate image quality and foraminal properties during a Spurling test under MRI acquisition. Ten asymptomatic individuals were included in the study (6 men and 4 women; age range 27 to 55 years). First, the subjects were positioned in the cervical compression device in a 3 T MRI scanner, and a volume T2 weighted (T2w) sequence was acquired in a relaxed supine position (3 min). Thereafter, the position and compressive forces on the patient’s neck (provocation position) were changed by maneuvering the device from the control room, with the aim to simulate a Spurling test, causing a mild foraminal compression, followed by a repeated image acquisition (3 min). A radiologist measured the blinded investigations evaluating cervical lordosis (C3–C7), foraminal area on oblique sagittal images and foraminal cross-distance in the axial plane. A total of three levels (C4–C7) were measured on the right side on each individual. Measurements were compared between the compressed and relaxed state. Reliability tests for inter- and intraclass correlation were performed. The device was feasible to use and well tolerated by all investigated individuals. Images of adequate quality was obtained in all patients. A significant increase (mean 9.4, p = 0.013) in the cervical lordosis and a decreased foraminal cross-distance (mean 32%, p < 0.001) was found, during the simulated Spurling test. The area change on oblique sagittal images did not reach a statistically significant change. The reliability tests on the quantitative measures demonstrated excellent intraobserver reliability and moderate to good interobserver reliability. Applying an individualized provocation test on the cervical spine, which simulates a Spurling test, during MRI acquisition was feasible with the novel device and provided images of satisfactory quality. MRI images acquired with and without compression showed changes in cervical lordosis and foraminal cross distance indicating the possibility of detecting changes of the foraminal properties. As a next step, the method is to be tested on symptomatic patients.

## Introduction

Degenerative cervical spinal disease is a common cause of pain with or without neurological symptoms, causing patient suffering and extensive interaction with the healthcare systems globally^1^. The panorama of symptoms can generally be categorized into three main groups; axial neck pain, radiculopathy, myelopathy or a combination of the three^2^. Cervical radiculopathy represents more or less specific symptoms including radiating pain in the upper extremities, with or without numbness and motor weakness, with a prevalence ranging between 107 and 580 per 100,000 individuals^3^. The underlying pathology is caused by changes in the tissue surrounding the nerve roots in the cervical foramina. These tissues, represented by lateral intervertebral disc herniations/bulgings, osteophytes, osteoarthritis of the facet joints or hypertrophy of the ligamentum flavum, can utilize a narrowing of the foramina causing compressive forces onto the spinal nerve roots^2^. Degenerative changes in the cervical spine are most frequently seen at level C5–C6, followed by C6–C7 and then C4–C5^4^.

The Spurling test is a commonly used clinical examination based on the premises of dynamic changes in the cervical foramina. The test is performed by extending the patient’s head, flexing it laterally with a slight rotation while an axial force (compression) is applied on the head^5^. A positive test is noted if the patient experiences pain radiating out in the ipsilateral arm. The Spurling test has been shown to have a high specificity of 0.89–1.00 (95% CI 0.59–1.00) and a moderate sensitivity ranging from 0.38 to 0.97 (95% CI 0.21–0.99)^6^. However, it can still be challenging to pin down the pain to a specific nerve root, especially considering the fact that there sometimes is a substantial dermatome overlap^7^. Another challenge in diagnostics is that the standard investigation includes relaxed supine MRI where the weight of the head is not taken into account as well as positions of the head that may aggravate the pain.

Therefore, our research group has developed a fully MRI compatible compression device that can apply controlled forces on the head and neck in different positions during image acquisition. The objective with the device was to enable MRI examination of the cervical spine in a provoked state by extending and bending the head laterally with a slight rotation and in addition slowly titrating up the forces applied, to simulate a clinical Spurling test. We believe that understanding cervical foraminal changes in different positions is essential to correctly identify and characterize neurological symptoms and to assist in the planning of individualized treatment strategies, both surgical and non-surgical. This type of provocation during MRI could possibly be a compliment to ordinary MRI, especially in patients with unclear pathology or when the diagnostics need to be strengthened.

The aim of this study was to test the feasibility of a new compression device and to evaluate image quality and foraminal properties during a Spurling test under MRI acquisition.

## Materials and methods

Magnetic resonance images were conducted with and without applied controlled compression of the cervical spine using the novel compression device in 10 healthy test subjects at Sahlgrenska University Hospital (Gothenburg, Sweden) between November and December 2020.

The individuals were recruited from the hospital staff after announcement. Inclusion criteria were: no present arm radiculopathy nor any cervical surgical procedures in the past. Prior periods of unexplained neck pain were accepted for inclusion. All procedures were performed in accordance with the Declaration of Helsinki and approved by the Regional Ethical Review Board in Gothenburg, Sweden (Dnr [574-18]). Written informed consent was signed by all attendees.

### The compression device

**Figure 1:**
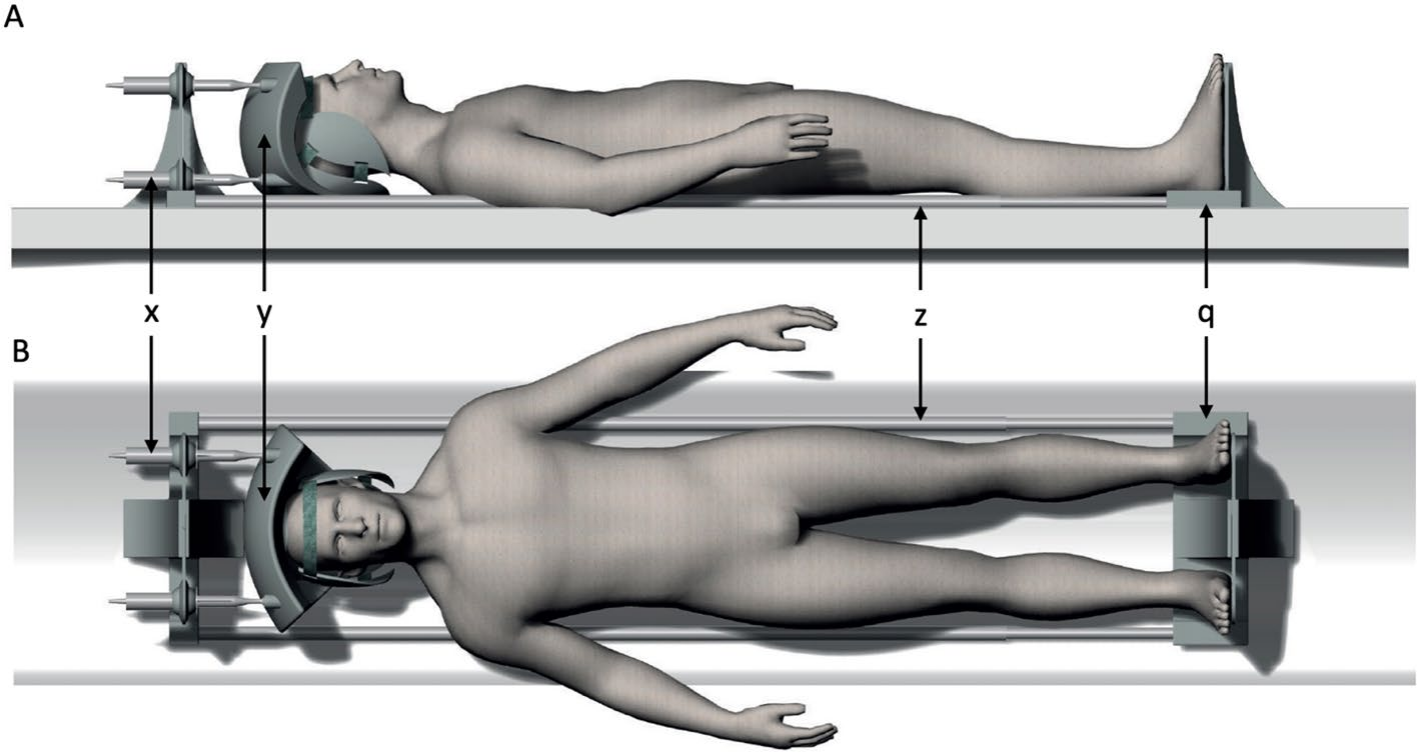
Graphic illustration of the cervical compression device with a positioned patient. (**A**) Sagittal view and (**B**) coronary view. The patient lay down in a supine position with the feet on a board (q) attached to the head part via two fiberglass rods (z). The head is strapped into a helmet-like construction (y) that is controlled by four individual hydraulic compression cylinders (x).

**Figure 2:**
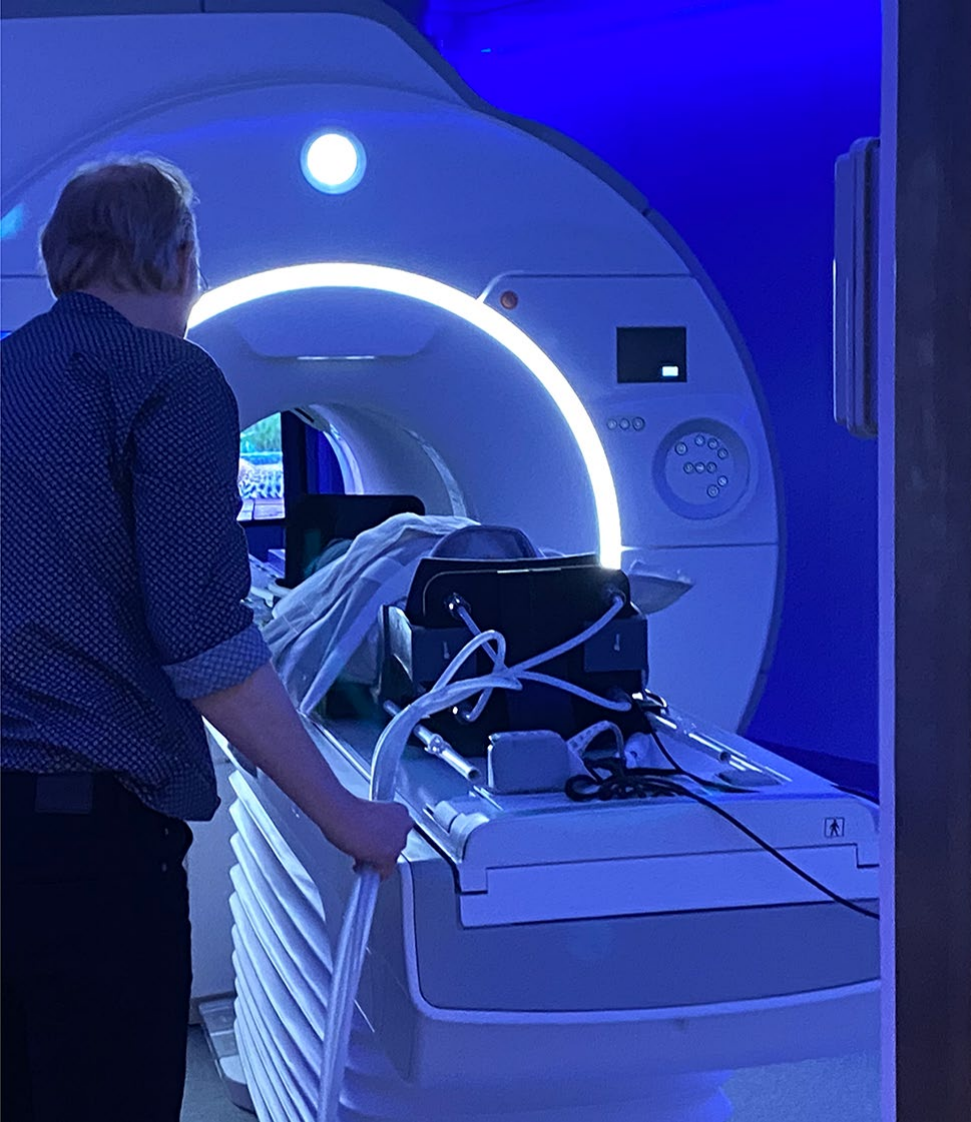
Picture showing a subject on the patient table ready to enter the MRI gantry.

Our research group has developed a fully MRI compatible compressive device (Patent no 2251201-6 pending); the Dynamic Magnetic Resonance Image Compression System (DMRICS) that can apply axial force via a water hydraulic system under real time force monitoring (Fig. 1). Since there is one cylinder in each corner of the head plate, the force can be applied individually, and the device consequently control sagittal extension, flexion, lateral flexion, and general axial compression. The forces can subsequently be applied on the head and neck in a supine position during an MRI session (Fig. 2). The person lay down in a supine position with the head positioned in a helmet and the feet on a footplate that can be adjusted and locked to adapt for different body lengths.

Maximum patient length is 2000 mm. The maximum BMI limit has not been tested but for persons below the maximum length, all BMI is assumed to be tolerated and the limiting factor is anticipated to be the size of the MRI gantry. The helmet and footplate are attached via fiberglass rods (in total 2300 mm long). The control board, which is not MRI compatible, needs to be located in the MRI control room. DMRICS is therefore equipped with long hoses to deliver the force through a water hydraulic system connected to the helmet, which transfers the forces to the cervical spine. On the control board, forces are induced by linear actuators. The forces are applied with 1 mm increments and monitored with a measuring equipment located on the control board to assure slow movements of the head/neck. All data is registered with our own developed software (DMRICS 1.0).

The part of the device used to position the patient for the MRI has a total weight of around 9 kg. To reduce discomfort and prevent movement-related distortions, particularly during pain provocation, we designed the loading equipment to be as comfortable as possible, e.g. hard materials in direct contact with the person were not used.

### Image acquisition and analysis

MRI was performed (GE 3 T Architect Medical Systems, Waukesha, WI, USA, DV.29) with a 3D (slice thickness of 1 mm and FOV25 × 25) T2-weighted (T2w) fast spin echo sequence, using an air coil. The test subjects had a first scan in the relaxed supine position directly followed by a second scan in the provoked position, simulating a Spurling test position. The machine placed the head in the position of extension, lateral deviation and a slight rotation and slowly applied a force on the head/neck until the subjects reported that they felt a clear pressure on the head in the direction planned. A new set of mages were acquired in the provoked position. The forces were applied by physically moving one fader per hydraulic cylinder on the control board.

The cervical neuroforamen has an exit angle of approximately 45 degrees ventrally^8^. It is of uttermost importance that the neuroforamen is observed from the correct and same angle to be able to compare measurements. Therefore, the T2w volume sequence (3D) enabled us to adjust the image plane accordingly.

One of the authors extracted single DICOM images representative of each foramina on the right side between C4 and C7 exiting nerve roots. The image in which the foramen appeared to be the narrowest was chosen for each C4–C7 foramina in the semi sagittal (approximately 45-degree angle from the midsagittal plane) and axial image planes. Sagittal images were also obtained to measure cervical lordosis.

To ensure blinded evaluation, the acquired MR images were individually coded and before extraction of quantitative estimates, sufficient image quality with low artefact appearance were ensured.

### Measurements and observers

**Figure 3:**
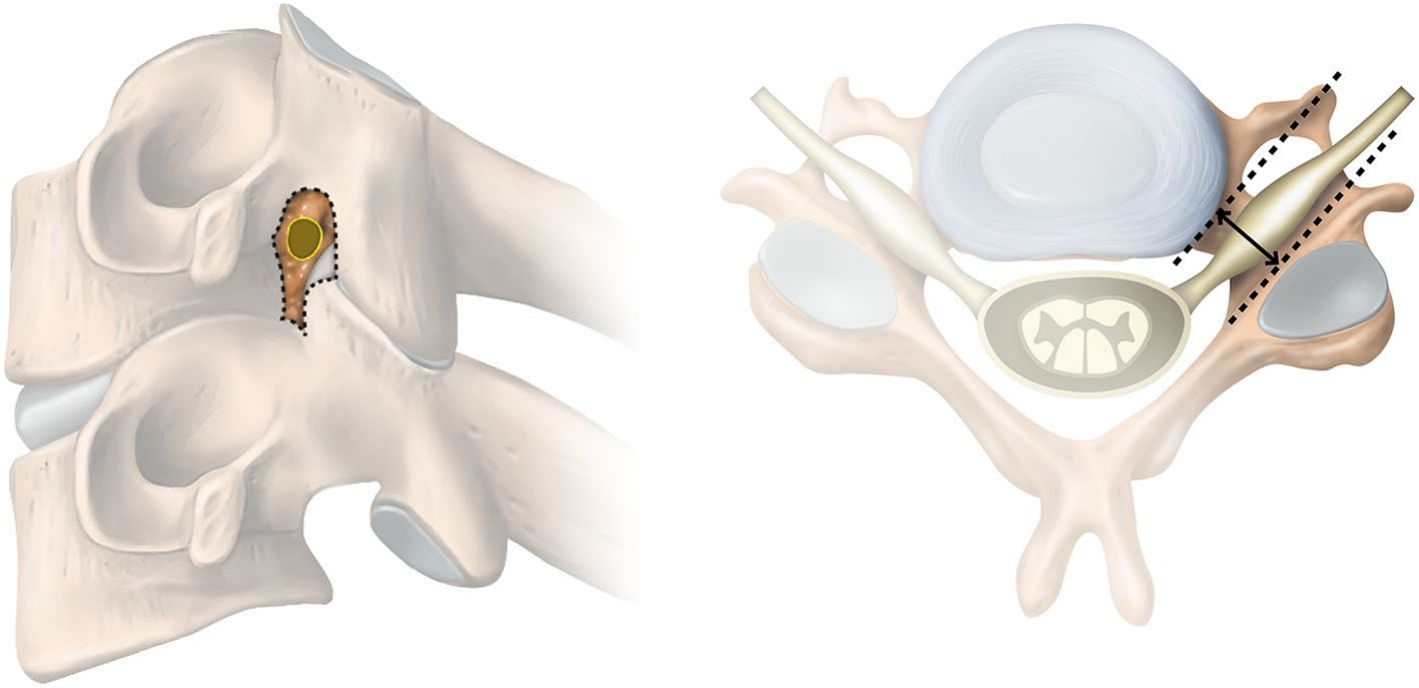
Foraminal area marked on the oblique sagittal image illustration to the left and the axial cross distance is marked on the illustration to the right.

A total of three foramina per individual (C4–C7) on the provoked (right for all subjects) side were measured. Three different quantitative measures were used including the lordosis angle between C3 and C7, the foraminal area on oblique sagittal images and the foraminal cross-distance on axial images (Fig. 3). RadiAnt DICOM Viewer 64-bit (2021.1-17805) was used for the measurements and the observers had an introduction to the software and practiced measurements on three other subjects prior to study start. Both relaxed and compressed images were blinded to the observers. To provide baseline information on the study population grading of disc degeneration was done according to Pfirrmann^9^ and qualitative foraminal compromise according to Park^10^ and Kim^11^. One radiologist performed all the readings twice with a four-month interval, to assess the intrarater reliability. A second radiologist performed measurements on five of the ten individuals (30 of 60 foramina) for calculation of interrater agreement.

### Statistical analysis

The collected data was found to be non-normally distributed and therefore we used Wilcoxon signed-rank test to detect statistically significant changes of quantitative continuous variables. Inter- and intraobserver agreement were analyzed using intra-class correlation coefficients (ICC). An ICC value less than 0.5 indicates poor reproducibility, ICC values 0.5–0.75 indicate moderate reliability, ICC values greater than 0.75–0.90 indicate good reliability, and values greater than 0.90 indicate excellent reliability^12^. Statistical analyses were performed using IBM SPSS software version 28.

### Informed consent

Featured individuals consented to image publication, understanding its purpose, usage, and potential impacts, including privacy. Consent records are kept and reviewable.

## Results

The healthy participants consisted of 6 men and 4 women: age range, 27 to 55 years. The discs in level C4–C7 were all scored as having a moderate degeneration (Pfirrmann grade 3 or 4). In 26/30 foramina no or mild foraminal stenosis on oblique MRI (Park grade 0 or 1) was found and 25/30 had no or mild stenosis on axial MRI (Kim grade 0 or 1).

### Feasibility of obtaining MRI using the device

All subjects were able to easily get in and out of the compressive device. These first ten healthy subjects undergoing MRI of the cervical spine in a compressed position all tolerated the compression well and did not express any claustrophobic issues. Further, the compressive device worked consistently in a predictable way for all ten subjects and relative force data (relative to the starting force) could be followed visually on the control board.

The acquisition time was approximately three minutes per volume sequence, which resulted in a total examination time of about 6 min including both relaxed and compressed position. All images were of good quality except for one foramina because of possible motion during image acquisition in the compressed state.

### Measured changes due to compression

**Figure 4:**
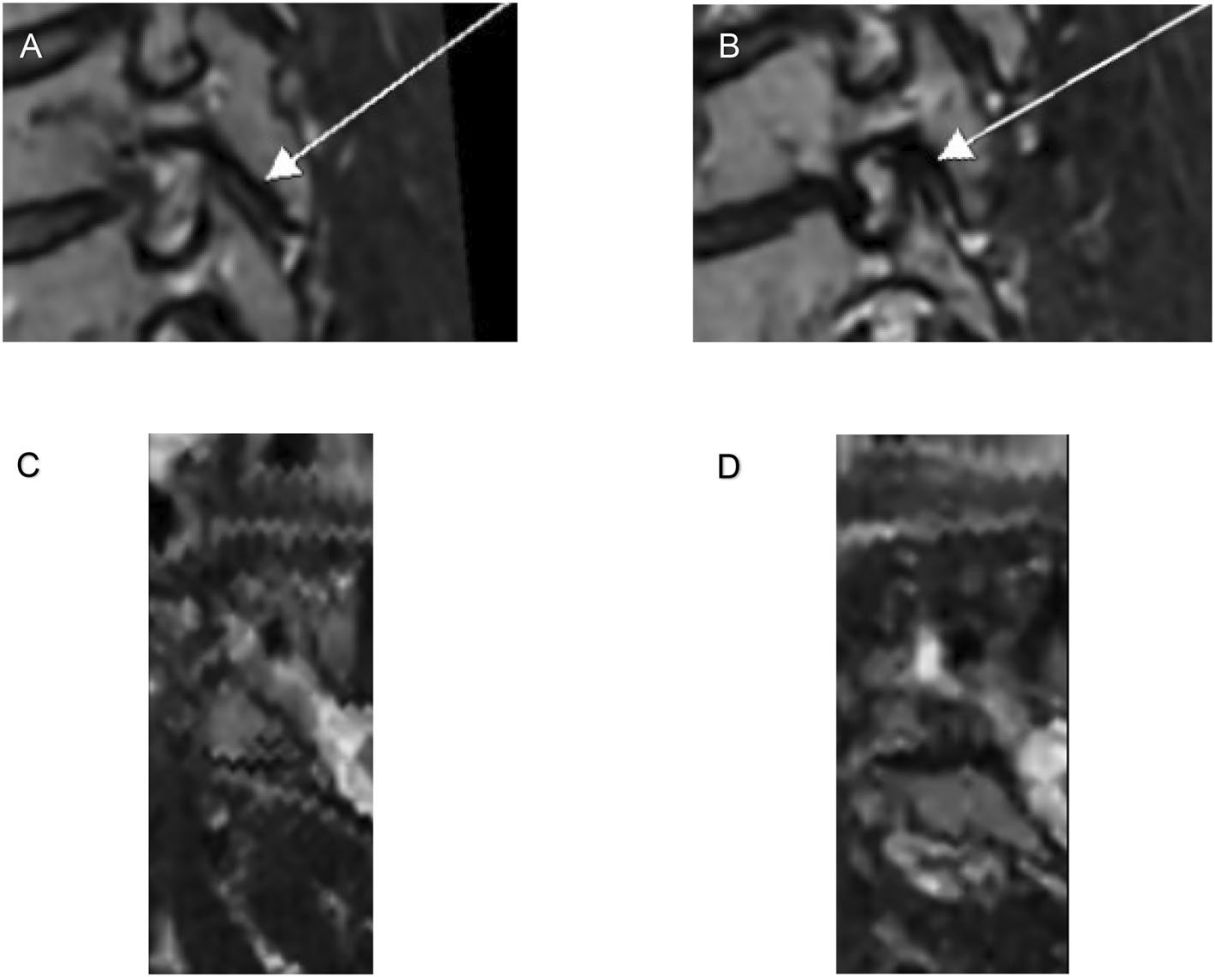
Magnetic resonance images of uncompressed and compressed foramina with the chosen sections demonstrating the smallest foraminal area/axial size for respective MR investigation. (**A,B**) depict the same subject and disc level before respectively after compression at level C4–C5 oblique sagittal plane, and in the axial plane (**C,D**).

Compression increased the lordosis angle with a mean of 9.4° (p = 0.013). We found that the foraminal cross-distance was significantly shorter, with a 32% (p < 0.001) reduction after compression, on a group level. The mean foraminal area measured on oblique semi sagittal images did not reach statistical significance (Table 1). The measured area increased for 15/29 foramina and decreased for 14/29 foramina. In all but one individual (who had foraminal area increase for all three levels), foramina with increase as well as decrease of the area were seen, indicating different effect on different foramina within an individual (Fig. 4).

**Table 1:** Differences in measurements between uncompressed and compressed MRI.

	Level	Mean				Difference		P		
	Disc level	Uncompressed		Compressed		Mean	%	P-value	CI (lower)	CI (upper)
			SD		SD					
Cervical angle (°)	C3–C7	12.290	9.600	21.690	8.020	9.400	176	0.013	3.770	15.029
Sagittal area (cm^2^)	C4–C7	0.277	0.104	0.259	0.131	0.018	− 6	0.581	− 0.049	0.013
Axial distance (mm)	C4–C7	2.590	0.993	1.749	0.573	0.841	− 32	< 0.001	− 1.189	− 0.494

*SD* standard deviation.

^a^Scale 0–5.

^b^Scale 0–3.

^c^Scale 0–2.

All MR images were comprehensive and available, with no missing or unavailable data. The artifact level was low in all images except in one, depicting one foramina in one patient. To reduce the influence of image artifacts on the evaluation, this image was excluded from further analysis and was reported as missing data.

### Intra- and interrater reliability

The intraobserver agreement for the quantitative measures were excellent and the interrater agreement good (for details see Table 2).

**Table 2:** Intra- and interobserver agreement. Observer A and B.

	A vs A	A vs B
ICC^a^		
Cervical angle	0.987	0.867
Sagittal area	0.991	0.652
Axial distance	0.981	0.616
k^b^		
Pfirrmann^c^	0.960	0.265
Park^d^	0.935	0.409
Kim^e^	0.977	0.461

^a^Intra Class Correlation Coefficient.

^b^Weighted Kappa.

^c^Scale 0–5.

^d^Scale 0–3.

^e^Scale 0–2.

## Discussion

This is the first study exploring the possibility to compare foraminal properties in the cervical spine on MRI with and without a controlled axial compression. The DMRICS was well tolerated and worked well on this pilot group during the MR image acquisition with good quality MR images obtained. Further, a decrease of axial foraminal cross-distance, an increased lordosis angle and a large variation of area changes on oblique sections was seen with the applied controlled compression.

To our knowledge no previous device in combination with MRI has been used to assist during image acquisition of the cervical spine during controlled compression, e.g., axial compression in combination with extension, lateral flexion or rotation. For the lumbar spine the Dynawell® has been used to apply axial compression and the effect on several spinal properties has been demonstrated^13^. Changes of foraminal measures have been demonstrated when obtaining images of the lumbar spine in different positions, revealing the dynamic range of effects achievable in the lumbar spine^14,15^. DMRICS, used in this cervical spine study, was developed for use in an MRI. Because the instrument to control the forces, as well as measuring the amount of load applied, was not MRI compatible, it had to be located outside the room of the MRI. This all worked well when tested in the actual MRI environment in the current pilot study.

Since high-resolution images without too long scanning time is a prerequisite for a good evaluation of the cervical foramina, it was important to have an optimized MR image acquisition protocol when applying controlled forces on the cervical spine using the DMRICS. Both the small size of the normal foramina and the fact that pain may affect the patient’s ability to stay still during examination complicates this process. Therefore, we used a volume sequence (3D) to assure that we could obtain an accurate image plane and angle for observing the foramina but also to minimize the scan time. The benefits of volume sequences have been previously established in lumbar and cervical spine^16,17^. However, there may also be disadvantages of using volume sequences, e.g., 3D T2w sequences of the cervical spine have been reported to show artifacts due to swallowing or vessel pulsation^18^. In our study some of the reformatted axial images were of somewhat less quality. Overall, the result here proved feasibility in obtaining MR images with sufficient quality using the DMRICS. Further good reliability was achieved for the measurements in this healthy study population.

The cross distance of the foramen on MRI showed a significant change after the controlled compression, with a mean of 32% reduction of cross distance while the change of area after compression did not reach statistical significance. In previous work by Bartlett et al. showed changes in cervical foraminal properties when comparing normal supine position with extension of the cervical spine. They graded the foraminal changes both using MRI, CT, and CT myelography and described narrowing of the foramina in extension^19^. Liu et al. demonstrated a significant increase of the foraminal area and foraminal heights when applying axial traction of different magnitudes during MRI acquisition^20^. Another study by Muhle et al. showed significant changes in foraminal size on oblique sagittal MRI with the head in extension, flexion, or axial rotation. Extension and ipsilateral rotation further denoted a decrease of foraminal size^21^. The difference between these studies compared to our study was that in none of the previous studies axial compression was included, nor alone or in combination with other movements (flexion, extension, rotation). It is important to understand that the changes of foraminal properties in flexion/extension in combination with compression is rather complex. Not only the size of the foramina (measured as area or a defined distance between landmarks) but also the shape of the foramina may change when exposed to different forces, which can directly affect the nerve root passing through. Since the present study was performed on individuals without radiculopathy, the changes that may occur with the change in position and compression of the neck, are not expected to be major. The foraminal area measurements showed both increased and decreased values after compression which may have to do with how the vertebras move in relation to each other during the applied force. Also, in the study by Takasaki et al*.*^22^ MRI of the cervical spine was obtained with the neck in a flexed, and rotated and slightly extended position, as in the present setting attempting to mimic the Spurling test. However, here without using a specific device controlling the forces, but instead with a fixed plate construct to hold the individual’s head in place. The study was, as ours, conducted in a healthy population. In accordance with the findings in our study, changes of different foraminal measurements were seen. The authors concluded that the Spurling test “produce functional, relevant, changes in the cervical intervertebral foramen, particularly in the midcervical spine”. This in contrast to our setting, where we were able to perform a fully controlled Spurling test with different loads and angles adapted to each individual as the test is performed in the clinic.

A relevant concern when inducing forces on the cervical spine is the potential harm it may cause to the anatomical structures. The current setting simulates a Spurling test, which is considered a standard physical exam in patients with intermittent radiculopathy. Performing this test on healthy individuals was considered having a very low risk of inducing a cervical injury.

The technique used in the present study may be of clinical value for patients where standard supine MRI is not sufficient to confirm the diagnosis of foraminal stenosis. We do not attempt to suggest our method as a first line in diagnosing cervical foraminal stenosis but as a complement in unclear cases. For example, there might be several foramina with moderate stenosis where the levels giving rise to the symptoms are unclear, which may influence the choice of adequate treatment^19^. The present approach to address foraminal pathology might sharpen the diagnostics in the clinical setting. The results in the present study with altered foraminal properties after applied controlled compression, but not always in a similar way, supporting previous findings^22^. The finding that some foramina increased and some decreased during the provocation is probably related to the reason that the Instantaneous Center of Rotation (ICR) of a spinal segment varies dependent on different factors, e.g. degree of disc degeneration^23^. This could indirectly affect the amplitude of the dynamics in each foramina.

Our method of course needs to be further investigated on patients with intermittent radiculopathy before clinical usefulness can be discussed. If the foramina of patients with intermittent radiculopathy will change differently in response to use of the DMRICS compared to the healthy subjects examined here, and if the patients will withstand such discomfort during an image acquisition without introducing to much motion artifacts to the MR images, remains to be tested.

## Limitations

The study focused on the feasibility of acquiring MRI in the desired provoked position using the novel device, justifying the study cohort. However, the small sample size of the cohort of normal subjects limits the value of the quantitatively measured foraminal changes.

Further, the measurable changes can not be generalized to any specific group of patients since the present investigation was performed in healthy individuals without the presence of radiculopathy or any neurological deficits.

## Conclusion

Applying individualized controlled compressive forces, trying to mimic a Spurling test, on the cervical spine during MRI acquisition is feasible with the novel device and provided images of satisfactory quality. The foraminal appearance during compression indicated altered foraminal properties that might inflict with passing nerve roots. Although the procedure worked effortlessly, future studies will have to stress the feasibility to use the device further in a real patient setting.

## Data Availability

The raw measurement data collected during the current study is available from the corresponding author on reasonable request.

## References

[1] Hoy D (2014). The global burden of neck pain: Estimates from the Global Burden of Disease 2010 study. Ann. Rheum. Dis..

[2] Rao R (2002). Neck pain, cervical radiculopathy, and cervical myelopathy: Pathophysiology, natural history, and clinical evaluation. J. Bone Jt. Surg..

[3] Mansfield M (2020). Cervical spine radiculopathy epidemiology: A systematic review. Musculoskel. Care.

[4] Radhakrishnan K (1994). Epidemiology of cervical radiculopathy: A population-based study from Rochester, Minnesota, 1976 through 1990. Brain.

[5] Jones SJ, Miller JMM (2022). Spurling Test.

[6] Thoomes EJ (2018). Value of physical tests in diagnosing cervical radiculopathy: A systematic review. Spine J..

[7] Lee MWL, McPhee RW, Stringer MD (2008). An evidence-based approach to human dermatomes. Clin. Anat..

[8] Kintzele L (2018). Oblique sagittal images prevent underestimation of the neuroforaminal stenosis grade caused by disc herniation in cervical spine MRI. Rofo Fortschritte auf dem Gebiete der Rontgenstrahlen und der Nuklearmedizin.

[9] Pfirrmann CWA (2001). Magnetic resonance classification of lumbar intervertebral disc degeneration. Spine.

[10] Park HJ (2013). A practical MRI grading system for cervical foraminal stenosis based on oblique sagittal images. Br. J. Radiol..

[11] Kim S (2015). A new MRI grading system for cervical foraminal stenosis based on axial T2-weighted images. Korean J. Radiol..

[12] Koo TK, Li MY (2016). A guideline of selecting and reporting intraclass correlation coefficients for reliability research. J. Chiropr. Med..

[13] Iwata T (2013). In vivo measurement of lumbar foramen during axial loading using a compression device and computed tomography. J. Spinal Disord. Tech..

[14] Zhong WMD (2015). In vivo dynamic changes of dimensions in the lumbar intervertebral foramen. Spine J..

[15] Hebelka H (2022). Axial loading during MRI induces lumbar foraminal area changes and has the potential to improve diagnostics of nerve root compromise. J. Clin. Med..

[16] Yamada H (2015). Improved accuracy of diagnosis of lumbar intra and/or extra-foraminal stenosis by use of three-dimensional MR imaging: Comparison with conventional MR imaging. J. Orthop. Sci..

[17] Kwon JW, Yoon YC, Choi SH (2012). Three-dimensional isotropic T2-weighted cervical MRI at 3T: Comparison with two-dimensional T2-weighted sequences. Clin. Radiol..

[18] Meindl T (2009). Magnetic resonance imaging of the cervical spine: Comparison of 2D T2-weighted turbo spin echo, 2D T2weighted gradient-recalled echo and 3D T2-weighted variable flip-angle turbo spin echo sequences. Eur. Radiol..

[19] Bartlett RJ (2012). MRI of the cervical spine with neck extension: Is it useful?. Br. J. Radiol..

[20] Liu J (2008). Quantitative changes in the cervical neural foramen resulting from axial traction: In vivo imaging study. Spine J..

[21] Muhle C (2001). In vivo changes in the neuroforaminal size at flexion-extension and axial rotation of the cervical spine in healthy persons examined using kinematic magnetic resonance imaging. Spine.

[22] Takasaki H (2009). The influence of cervical traction, compression, and spurling test on cervical intervertebral foramen size. Spine.

[23] Guo Z (2019). Clinical relevance of cervical kinematic quality parameters in planar movement. Orthop. Surg..

